# Major Dietary Patterns in Relation to Stunting among Children in Tehran, Iran

**DOI:** 10.3329/jhpn.v31i2.16384

**Published:** 2013-06

**Authors:** Fatemeh Esfarjani, Roshanak Roustaee, Fatemeh Mohammadi-Nasrabadi, Ahmad Esmaillzadeh

**Affiliations:** ^1^Department of Food Policy and Nutrition Planning, National Nutrition and Food Technology Research Institute, Faculty of Nutrition Sciences and Food Technology, Shahid Beheshti University of Medical Sciences, Tehran, Iran;; ^2^Food Security Research Center,; ^3^Department of Community Nutrition, School of Nutrition and Food Science, Isfahan University of Medical Sciences, Isfahan, Iran

**Keywords:** Child, Dietary pattern, Factor analysis, Stunting, Iran

## Abstract

To the best of our knowledge, no information is available to link major dietary patterns to stunting during childhood, although dietary patterns are associated with chronic diseases. This study was conducted to determine the relationship between major dietary patterns and stunting in the first grade pupils of Tehran in 2009. In this case-control study, 86 stunted children (defined as height-for-age of less than the 5th percentile of CDC2000 cutoff points) were enrolled from among 3,147 first grade pupils of Tehran, selected using a multistage cluster random-sampling method. Participants for the control group (n=308) were selected randomly from non-stunted children (height-for-age more than the 5th percentile of CDC2000 cutoff points), after matching for age, sex, and area of residence. Dietary data were collected using two 24-hour dietary recalls through face-to-face interview with mothers. Factor analysis was used for identifying major dietary patterns. Mean consumption of dairy products (308±167 vs 382±232 g/day, p<0.05), dried fruits and nuts (2.58±9 vs 7.15±26 g/day, p<0.05) were significantly lower among stunted children than those in the control group. Three major dietary patterns were identified: ‘traditional dietary pattern’ that was dominated by bread, potato, fats, eggs, flavours, vegetables other than leafy ones, sugar, drinks, and fast food; ‘mixed dietary pattern’ that was dominated by leafy vegetables, fast foods, nuts, fats, cereals other than bread, fruits, legumes, visceral meats, sugars, eggs, and vegetables other than leafy vegetables; and ‘carbohydrate-protein pattern’ that was dominated by sweets and desserts, poultry, dairy, fruits, legumes, and visceral meats. No significant relationships were found between traditional and mixed dietary patterns and stunting. Individuals in the third quartile of carbohydrate-protein dietary pattern were less likely to be stunted compared to those in the bottom quartile (OR: 0.31, 95% CI 0.13-0.78, p<0.05). Adherence to dietary patterns high in protein (e.g. dairy, legumes, and meat products) and carbohydrates (e.g. fruits, sweets, and desserts) might be associated with reduced odds of being stunted among children.

## INTRODUCTION

Stunting, defined as “the gaining of insufficient height relative to age” (1) during childhood is a major public-health problem in underdeveloped and developing countries (1-4). The 2000 report of the World Health Organization (WHO) stated that 215 million children were stunted. Findings from a national survey in Iran in 2005 indicated that 4.7% of Iranian children were affected (5). Stunting in children is associated with current, and possibly later, delayed mental and motor development (6-7). Limited work-capacity due to reduced muscle mass has also been reported among stunted people (6). Earlier studies have also found increased obstetric risks among stunted women (8).

Linear growth retardation, which is the manifestation of chronic malnutrition, can be developed from inadequate intake of food, inappropriate quality of diet, or a combination of both (6-10). Numerous studies that have been conducted on growth-limiting nutrients have indicated that primary deficiency of zinc, vitamin A, and iron, along with insufficient intake of protein and energy, might result in stunting (5,11-14). However, it must be kept in mind that interactions between nutrients might confound the association of a specific nutrient with stunting. To overcome these problems, nutritional epidemiologists have suggested the use of a dietary pattern approach as a new direction in nutritional epidemiology to find diet-disease relationships (14). Using a multivariate approach could control potential dietary confounders and food and nutrient interactions (15-17).

Generally, several studies have assessed dietary patterns in relation to numerous outcomes. Most of these studies have been performed among adults in western societies; limited information is available about major dietary patterns of children worldwide, particularly in region of the Middle East not studied well. Furthermore, although the association of several nutrients with stunting has received great attention, we are unaware of any studies linking major dietary patterns to stunting, particularly among children (12-14,18). This study was, therefore, conducted to determine the relationship between major dietary patterns and stunting in the first grade pupils of Tehran, Iran.

## MATERIALS AND METHODS

### Subjects

This case-control study has been done in the framework of a cross-sectional survey that was performed among 3,147 first grade pupils aged 7 years, who were selected using a multistage cluster random-sampling method from 42 elementary schools in 5 districts of Tehran (northern, southern, eastern, western, and central part of Tehran). Children's weight was measured while the subjects were minimally clothed without shoes, using digital scales and recorded to the nearest 100 g. Height was measured in a standing position, without shoes, using tapemeter while shoulders were in a normal state. Body mass index was calculated as weight in kilogramme divided by height in metres squared. To avoid subjective error, all measurements were taken by the same person. Stunting was defined as height-for-age of less than the 5th percentile of the Center for Disease Control and Prevention 2000 (CDC2000) cutoff points. Qualified children (N=117), whose parents agreed to participate in the study, were specified as a case group (n=86). After matching for age, sex, and area of residence, we selected 3 apparently healthy non-stunted children [(height-for-age equal to and more than the 5th percentile of CDC2000 cutoff points for each stunted kid as their controls (n=308)]. Therefore, in the current study, we examined 394 students in total.

The Ethics Committee of the National Nutrition and Food Technology Research Institute approved this study, and informed written consent was obtained from all participants and their parents.

### Assessment of dietary intake

Dietary intakes of the study participants were assessed by means of two quantitative non-consecutive 24-hour dietary recalls (one for a weekday and the other for a weekend). Subjects were usually interviewed, along with their parents by a face-to-face method. The subjects and their parents were asked to recall all foods and beverages they consumed during the preceding 24-hours. To assist subjects and their parents to recall accurately, household utensils were used. Portion-sizes of consumed foods were converted to gramme, using household measures. Each food and beverage was then coded according to prescribed protocol and analyzed for contents of energy and other nutrients, using Nutritionist-III (N3) Software program designed for Iranian foods. Almost all foods eaten by our subjects could be coded. When a particular ethnic food was not in the database of N3, we coded it as a similar item.

### Assessment of other variables

Required information on demographic characteristics and socioeconomic status (household-size, parity, parent's job, education, marital status, mother's age, and home ownership), birthweight and height, and duration of breastfeeding was obtained for each subject through interviewing their mothers.

### Statistical analysis

We used factor analysis to identify major dietary patterns. First, due to the large number of food items relative to the number of participants, we assigned each food-item into 22 predefined food groups (Table 1), based on the similarity in their nutrient contents. Principal component analysis was done with the factors rotated by orthogonal transformation. The natural interpretation of the factors in conjunction with eigenvalues (≥1.3) and Scree plot determined whether a factor should be retained. The derived factors (dietary patterns) were labelled on the basis of our interpretation of data and on prior literature. The factor score for each pattern was calculated by summing intakes of food groups weighted by their factor loadings, and each participant received a factor score for each identified pattern.

**Table 1.** Food grouping used in the dietary pattern analysis

Food group	Food item
Bread	Dark breads (Iranian), barley bread, bulgur, white breads (lavash, baguettes), toasted bread, sweet bread
Other cereals	White flour, noodles, pasta, rice, popcorn, cornflakes
Legumes	Beans, peas, lima beans, broad beans, lentils, soy
Potato	Potatoes, French fries, potato chips
Leafy vegetables	Spinach, lettuce, mixed vegetables
Other vegetables	Carrots, cabbage, cauliflower, kale, tomatoes, cucumber, eggplant, celery, green peas, green beans, green pepper, turnip, corn, squash, mushrooms, onions, garlic, okra, beats
Fruits	Fruit pears, apricots, cherries, apples, raisins or grapes, bananas, cantaloupe, watermelon, oranges, grapefruit, kiwi, strawberries, peaches, nectarine, tangerine, mulberry, plums, persimmons, pomegranates, lemons, pineapples, figs and dates, apple juice, orange juice
Red meats	Beef, lamb, hamburger
Poultry	Chicken with or without skin
Fish	Canned tuna fish, other fish
Visceral meats	Beef liver
Eggs	Eggs
Dairy products	Milk (skimmed or low-fat/high-fat, whole); chocolate milk, yogurt (low-fat/high-fat/cream); yogurt drink (doogh), cream cheese, other cheeses, ice cream
Fat and oil	Butter, margarine, olive, olive oils, hydrogenated fats, animal fats, vegetable oils, mayonnaise, cream
Sugars	Sugars, candies, gaz (an Iranian confectionery item made of sugar, nuts, and tamarisk), condiments jam, jelly, honey
Sweets and desserts	Biscuits, cakes, cookies, chocolates
Nuts	Peanuts, almonds, pistachios, hazelnuts, roasted seeds, walnuts
Dried fruits	Dried figs, dried mulberries, other dried fruits, raisins
Drinks	Tea, coffee, soft drinks, synthetic fruit juice
Flavours	Tomato sauce, tomato pasta, pickles, vinegar, salty cucumber, pomegranate paste
Fast food	Sausages, salami, bologna, pizza, kebab, lasagna
Others	Gum, crackers

Participants were categorized based on quartiles of dietary pattern scores. To determine the association of dietary patterns with stunting, we used multivariate logistic regression. Regression analysis was controlled for their age, sex, parent's age and education, birthweight, family-size, duration of breastfeeding, and total energy intake in different models. The first quartile of dietary pattern scores was reconsidered a reference in each model. All statistical analyses were done by Statistical Package for Social Sciences software (SPSS Inc, version 16).

## RESULTS

For the whole population (n=3,147) in the cross-sectional survey, the prevalence of stunting was 3.7%—significantly higher in girls than that in boys (4.4% vs 2.8%, p<0.05). Also, the mean birthweight was significantly lower in stunted children (2.9±0.6 kg vs 3.2±0.5 kg, p<0.05).

Dietary intakes of stunted and non-stunted children are provided in Table 2. We found no significant difference in total energy intake as well as macro- and micronutrients intake between stunted and non-stunted children. However, stunted kids had slightly lower intakes of calcium compared to their non-stunted counterparts (p=0.06). Dietary intakes of dairy products, dried fruits, and nuts were lower but fat and oil consumption was higher among stunted children compared to the non-stunted group (Table 3). The difference in the consumption of bread, poultry, visceral meats as well as sweets and desserts between cases and controls was nearly significant.

**Table 2.** Dietary intakes by study participants

Dietary intake	Mean±SD		p value
	Cases (n=86)	Controls (n=308)	
Foods and food groups (g/day)			
Bread	72±60	87±64	0.05
Other cereals	12±35	16±41	0.39
Legumes	43±71	31±47	0.14
Potato	20±28	23±36	0.52
Leafy vegetables	41±100	32±48	0.4
Other vegetables	121±120	123±116	0.92
Fruits	339±287	343±316	0.91
Red meats	56±88	49±68	0.50
Poultry	25±40	35±43	0.05
Fish	14±49	9±29	0.33
Visceral meats	0.2±2	1±8	0.06
Eggs	23±30	24±30	0.93
Dairy products	308±167	383±232	<0.05
Fat and oil	38±29	31±22	0.04
Sugars	21±14	22±20	0.44
Sweets and desserts	52±51	63±58	0.09
Nuts	8±17	11±23	0.13
Dried fruits	3±9	7±26	0.01
Drinks	238±198	243±178	0.84
Flavours	5±15	6±17	0.48
Fast foods	202±127	201±113	0.91
Others	17±29	20±30	0.51
Nutrients			
Energy (kcal/day)	2104±858	2178±77	0.46
Protein (g/day)	75±37.6	79±35	0.32
Fat (g/day)	77±35	75±30	0.67
Carbohydrate (g/day)	297±137	317.4±131	0.22
Calcium (mg/day)	930±457	1034±437	0.06
Iron (mg/day)	26±20	26±19	0.79
Zinc (mg/day)	10±6	11±5	0.26
Vitamin A (mcg/day)	690±815	671±668	0.37
Vitamin C (mg/day)	110±122	106±86	0.30

Factor loadings of foods and food groups in major dietary patterns are presented in Table 3. Three dietary patterns were identified: ‘traditional dietary pattern’ that was high in bread, potato, fats, eggs, flavours, vegetables other than leafy vegetables, sugar, drinks, and fast foods; ‘mixed dietary pattern’ that was dominated by leafy vegetables, fast foods, nuts, fats, cereals other than bread, fruits, legumes, visceral meats, sugar, eggs, and other vegetables; and ‘carbohydrate-protein dietary pattern’ that was greatly dominated by sweets and desserts, poultry, dairy, fruits, legumes, and visceral meats. On the whole, these dietary patterns explained 23.1% of the whole variance.

**Table 3.** Factor loadings of food groups in major dietary patterns^1^

Food group	Major dietary patterns		
	Traditional	Mixed	Carbohydrate- protein
Bread	0.654	-	-
Potato	0.556	-	-
Fat and oil	0.520	-	-
Eggs	0.498	-	-
Flavours	0.451	-	-
Sugar	0.300	-	-
Drinks	0.264	-	-
Leafy vegetables	-	0.696	-
Other vegetables	0.409	-	-
Fast foods	-	0.567	-
Nuts	-	0.392	-
Other cereals	-	0.323	-
Legumes	-	0.296	-
Visceral meats	-	0.289	-
Sweets and desserts	-	-	0.583
Poultry	-	-	0.472
Dairy products	-	-	0.462
Fruits	-	-	0.397
Others	-	-	0.343
Red meats	-	-	-
Fish	-	-	-
Dried fruits	-	-	-
Percentage of variance explained	8.9	7.7	6.5

^1^Factor loadings of less than 0.2 have been omitted for simplicity

Characteristics of the study participants across quartiles of major dietary patterns are shown in Table 4. Mean age, birthweight, and family-size were not significantly different across quartiles of dietary pattern scores. Individuals in the highest quartile of different major dietary patterns were not significantly different in terms of parental education and prevalence of stunting compared to those in the lowest quartile.

**Table 4.** Characteristics of participants and their dietary intakes across quartiles of dietary pattern scores

Characteristics	Traditional pattern's score		p	Mixed pattern's scores		p	Carbohydrate-protein pattern's score		p
	Q1	Q4		Q1	Q4		Q1	Q4	
Age (months)	82±4	82±4	0.47	82±4	83±4	0.42	83±4	82±4	0.58
Girls (%)	71	58	0.15	55	67	0.28	67	57	0.44
Mother's age at delivery (years)	26±5	27±5	0.26	27±5	26±5	0.59	28±6	27±4	0.23
Birthweight (g)	3±1	3±1	0.51	3±1	3±1	0.73	3±1	3±1	0.65
Family-size	4±1	4±1	0.56	4±1	4±1	0.55	4±1	4±1	0.43
Mother's education			0.40			0.41			0.17
No education	44	0		22	44		0	33	
Primary	23	35		16	39		26	29	
Secondary and under diploma	23	26		29	20		30	26	
Diploma and university	55	44		56	51		33	52	
Fathers' education			0.75			0.54			0.94
No education	40	20		20	20		0	40	
Primary	23	37		27	33		23	30	
Secondary and under diploma	23	25		29	24		26	25	
Diploma and university	47	45		48	59		51	48	
Stunting (%)	16	25	0.14	22	25	0.11	20.2	27	0.32

Odds ratios for stunting across quartiles of dietary patterns are provided in Table 5. We found no significant associations between ‘traditional’ and ‘mixed’ dietary patterns and stunting either before or after adjustment for potential confounders. Individuals in the third quartile of ‘carbohydrate-protein’ dietary pattern were less likely to be stunted compared to those in the lowest quartile (OR: 0.4, 95% CI 0.18-0.88). The associations strengthened after further controlling for potential confounders and total energy intake (OR: 0.31, 95% CI 0.13-0.78).

**Table 5.** Multivariate-adjusted odds ratios and 95% confidence intervals for stunting across quartiles of dietary pattern scores^1^

Dietary pattern	Quartiles of dietary pattern's scores			
	1 (Lowest)	2	3	4 (Highest)
Traditional dietary pattern				
Crude	1.00	0.69 (0.35-1.36)	0.73 (0.37-1.44)	0.65 (0.33-1.30)
Model 1	1.00	0.69 (0.35-1.36)	0.74 (0.37-1.45)	0.65 (0.33-1.30)
Model 2	1.00	0.77 (0.36-1.65)	0.72 (0.33-1.54)	0.79 (0.37-1.67)
Model 3	1.00	0.79 (0.36-1.70)	0.75 (0.33-1.71)	0.87 (0.34-2.23)
Mixed dietary pattern				
Crude	1.00	1.05 (0.53-2.10)	1.11 (0.56-2.21)	0.94 (0.46-1.89)
Model 1	1.00	1.05 (0.53-2.11)	1.12 (0.56-2.24)	0.95 (0.46-1.92)
Model 2	1.00	1.18 (0.54-2.57)	1.45 (0.66-3.17)	0.92 (0.41-2.04)
Model 3	1.00	1.18 (0.54-2.57)	1.46 (0.67-3.20)	0.96 (0.42-2.19)
Carbohydrate-protein dietary pattern				
Crude	1.00	1.42 (0.75-2.70)	0.40 (0.18-0.88)*	0.73 (0.36-1.46)
Model 1	1.00	1.43 (0.75-2.71)	0.40 (0.18-0.89)*	0.73 (0.36-1.46)
Model 2	1.00	1.53 (0.74-3.19)	0.31 (0.13-0.77)*	0.93 (0.42-2.06)
Model 3	1.00	1.54 (0.74-3.20)	0.31 (0.13-0.78)*	0.96 (0.42-2.18)

^1^Model 1: Adjusted for age and sex; Model 2: Additionally adjusted for mother's age, parental education, birthweight, duration of breastfeeding, and family-size; Model 3: Further adjusted for total energy intake

## DISCUSSION

We found a significant protective association between adherence to ‘carbohydrate-protein’ pattern and stunting among the first grade students in Tehran. This association remained significant even after taking potential confounders into account. Overall, no significant associations were observed between ‘traditional’ and ‘mixed’ dietary patterns and stunting in this population. To the best of our knowledge, this study is the first examining the association between major dietary patterns and stunting.

Although stunting is still highly prevalent in underdeveloped and developing countries, few studies have assessed the role of whole diet in its aetiology. Earlier studies on dietary determinants of stunting have mostly focused on nutrients (19). Findings from such studies have indicated that deficiency of zinc, vitamin A, and iron as well as inadequate intake of protein and energy is the most important nutritional cause of stunting (5,11-13,20). In the current study, we found no significant differences in dietary intakes of energy and other nutrients, including calcium by comparing stunted and non-stunted children. Due to the age difference and the use of different sets of diets and environments, our results are not directly comparable with those of earlier studies. The discrepant findings might be explained by the different study designs. In terms of foods and food groups intake, we found that stunted children had significantly lower intake of ‘dairy products’ and ‘nuts and dried fruits’ and slightly higher intake of ‘fats’. Based on these findings, it seems that the diet quality differs between cases and controls (21-24). Calcium has been mainly provided by consumption of milk and dairy foods in the control group and by consumption of foods from other dietary sources, like green leafy vegetables and legumes, in case group. Besides, calcium, milk, and dairy products contain other necessary nutrients, like protein, for promoting growth in height. Therefore, it can be concluded that not only the amount of total calcium intake but also its dietary sources containing protein contents might affect height. Ibrahim and colleagues in Egypt made similar conclusions that deficiency of several nutrients, including proteins, is seen in stunted children, and the combined effect of these deficiencies might have a role in the retardation of growth in height (5). To detect the effect of this combined effect, the ‘dietary pattern’ approach might be more relevant to dietary aetiology of stunting than that of nutrients (14).

The dietary pattern approach that includes food behaviours of individuals may provide greater information on nutritional aetiology of stunting. Using such a multivariate approach would overcome the problems of collinearity among nutrients, unknown dietary confounders as well as interactions between foods and nutrients. In addition, based on this holistic approach, dietary interventions will be easier and more thorough (16,25).

In the current study, we identified three major dietary patterns, among those, the one with greater amounts of carbohydrate and protein was inversely related to stunting, even after statistical controlling for potential confounders. We didn't find any association between dietary patterns and stunting to compare our findings. However, our results are in line with previous studies, indicating that adequate intake of proteins is required for normal growth. We did not find any association between ‘traditional’ and ‘mixed’ dietary patterns and stunting. Existence of healthy and unhealthy foods together in these dietary patterns might be the reason for lack of association. On the whole, results of our dietary pattern analysis are in the same direction with earlier studies on the significant difference in dairy consumption between stunted and non-stunted children. As stunting is an indicator of long-term malnutrition, monitoring the growth and nutritional status of children since birth should be considered to prevent stunting and its adverse consequences.

### Strengths and Limitations

Several limitations must be considered in the interpretation of our findings. First, the case-control design of this study did not allow us to infer causality. Therefore, the exact association between major dietary patterns and stunting must be confirmed in prospective studies. It must, however, be kept in mind that appropriate analysis of case-control data represent a valuable initial step in identifying diet-disease relations. Moreover prospective cohort studies have their own limitations. Second is the reliance on two days of dietary recalls as a measure of usual dietary intake. The 24-hour recall method is susceptible to recall bias, both for identification of foods eaten and for quantification of portion-sizes. Collecting dietary data by highly-trained interviewers in this study reduce this type of error. Although recalling two days cannot cover all day-to-day variations in dietary intake, using non-consecutive days extends their coverage. Furthermore, various amount estimation tools, including common sizes of mugs, spoons and glasses were used in collecting more accurate data. The 24-hour dietary recall method is the most commonly-used method for dietary surveys. As this method is based on actual intake, it could be used in estimating absolute rather than relative intake of nutrients. This method is completely open-ended, so it can accommodate any food or food combination reported by the subject. This could not be obtainable, using a limited number of food-items in a structured questionnaire. Recalls are specifically useful for estimating dietary intakes of culturally-diverse populations representing a wide range of foods and eating habits. In addition, other epidemiologic studies, such as the Ten State Nutrition Survey, NHANES I, and the Multiple Risk Factor Intervention Trial, have also used the recall method for gathering dietary data. Collecting dietary data for two days could provide more accurate estimates of dietary intake compared to just for one day. The validity of data provided by this method has been reported previously, and it has been shown that estimates obtained from recalls are comparable to those obtained with more precise methods, such as dietary records (26). However, as with all dietary assessment tools, the existence of recall bias, under- and over-reporting of dietary intakes as well as lack of coverage of seasonal variation by this method cannot be excluded. Collecting dietary data by highly-trained interviewers in this study reduced this type of error. Although recalling two days cannot cover all day-to-day variations in dietary intake, using non-consecutive days or consecutive 7 days extends their coverage. Finally, stunting is a heterogeneous multi-factorial disorder and besides dietary factors, other variables, such as hereditary factors and metabolic conditions must be considered.

### Conclusions

We found that adherence to dietary patterns high in protein (e.g. dairy, legumes, and meat products) and carbohydrates (e.g. fruits, sweets, and desserts) might be associated with reduced odds of being stunted among children. Further studies are required to confirm our findings.

## ACKNOWLEDGEMENTS

The authors would like to appreciate the Research Council of National Nutrition and Food Technology Research Institute for their financial support. The supports from school teachers, principal, students, and their parents are also highly appreciated. We also thank Seyedeh Marjan Khalafi and Seyed Mohammad Hosseini for their help.
